# Development of a melting-curve based multiplex real-time PCR assay for simultaneous detection of *Streptococcus agalactiae* and genes encoding resistance to macrolides and lincosamides

**DOI:** 10.1186/s12884-018-1774-5

**Published:** 2018-05-03

**Authors:** Eliane Saori Otaguiri, Ana Elisa Belotto Morguette, Alexandre Tadachi Morey, Eliandro Reis Tavares, Gilselena Kerbauy, Rosângela S. L. de Almeida Torres, Mauricio Chaves Júnior, Maria Cristina Bronharo Tognim, Viviane Monteiro Góes, Marco Aurélio Krieger, Marcia Regina Eches Perugini, Lucy Megumi Yamauchi, Sueli Fumie Yamada-Ogatta

**Affiliations:** 1 Departamento de Microbiologia, Centro de Ciências Biológicas, Universidade Estadual de Londrina, Londrina, Paraná Brazil; 2 Departamento de Enfermagem, Universidade Estadual de Londrina, Londrina, Paraná Brazil; 3 Laboratory of Bacteriology, Epidemiology Laboratory and Disease Control Division, Laboratório Central do Estado do Paraná – LACEN, Curitiba, PR Brazil; 4 Departamento de Medicina, Hospital Universitário de Maringá, Universidade Estadual de Maringá, Maringá, Brazil; 5 Departamento de Ciências Básicas da Saúde, Universidade Estadual de Maringá, Maringá, Brazil; 6 Instituto Carlos Chagas, Fundação Instituto Oswaldo Cruz, Curitiba, Brazil; 7 Departamento de Patologia, Análises Clínicas e Toxicológicas, Centro de Ciências da Saúde, Universidade Estadual de Londrina, Londrina, Paraná, Brazil; 8 Departamento de Microbiologia, Universidade Estadual de Londrina, Centro de Ciências Biológicas, Rodovia Celso Garcia Cid, PR 445, km 380. CEP, Londrina, 86057-970 Brazil

**Keywords:** *cfb* gene, *erm* and *mef* antimicrobial resistance markers, Group B *Streptococcus*, Melting curve, Pregnant women; vaginal-rectal swab

## Abstract

**Background:**

*Streptococcus agalactiae* or Group B *Streptococcus* (GBS) remains the leading cause of infections in newborns worldwilde. Prenatal GBS screening of pregnant women for vaginal-rectal colonization is recommended in many countries to manage appropriate intrapartum antimicrobial prophylaxis for those identified as carriers. In this study, a novel melting-curve based multiplex real-time PCR assay for the simultaneous detection of GBS and macrolide and lincosamide resistance markers was developed. The usefulness of the assay was evaluated for rapid and accurate prenatal GBS screening.

**Methods:**

One hundred two pregnant women who were at 35–37 weeks of gestation were enrolled in this study. The analytical performance of the multiplex real-time PCR was first tested using a panel of reference and clinical bacterial and fungal strains. To test the clinical performance, vaginal-rectal swabs were obtained from pregnant women who were seen at the teaching hospital for regular prenatal care. The results of real-time were compared with those obtained from microbiological analyses.

**Results:**

The real-time PCR assay showed 100% specificity and a limit of detection of 10^4^ colony forming units equivalent per reaction. The prevalence of GBS colonization among the population studied was 15.7% (16/102) based on a positive culture and the real-time PCR results. Agreement between the two assays was found for 11 (68.75%) GBS colonized women. Using the culture-based results as a reference, the multiplex real-time PCR had a sensitivity of 91.7% (11/12, CI 59.7–99.6%), a specificity of 95.5% (86/90, CI 89.8–98.7%), a positive predictive value of 73.3% (11/15, CI 44.8–91.1%) and a negative predictive value of 98.9% (86/87, CI 92.9–99.9%).

**Conclusion:**

The multiplex real-time PCR is a rapid, affordable and sensitive assay for direct detection of GBS in vaginal-rectal swabs.

**Electronic supplementary material:**

The online version of this article (10.1186/s12884-018-1774-5) contains supplementary material, which is available to authorized users.

## Background

*Streptococcus agalactiae* or Group B *Streptococcus* (GBS) is a leading cause of infections in newborns worldwide [1, 2]. Neonatal GBS diseases are associated with significant morbidity and mortality, and infants who survive may incur long-term disabilities [3, 4]. GBS can asymptomatically colonize the human gastrointestinal and/or genital tract [5–7]. During pregnancy, this colonization represents the most important risk factor for the development of invasive GBS diseases, most of which affect babies within the first week of life [8]. Maternal GBS transmission to the newborn may occur vertically by ascending infection or during passage through the birth canal [9].

Women can be transiently, intermittently or persistently colonized by GBS in their vaginal or anorectal mucosae [6]. Accordingly, the risk of maternal GBS transmission to the newborn and development of infection persists. The prevention strategy based on bacterium screening and intrapartum antimicrobial prophylaxis (IAP) in those pregnant women identified as carriers has led to a substantial reduction in the incidence of neonatal GBS diseases in various regions of the world [10]. Currently penicillin is recommended as first-line antibacterial for IAP, and clindamycin or erythromycin (second line) may be used in penicillin-allergic pregnant women at risk of anaphylaxis [8]. In general, GBS isolates remain susceptible to penicillin [5, 11] however isolates with reduced susceptibility to this antibacterial have been reported [12]. In contrast, resistance to clindamycin and erythromycin among GBS isolated from pregnant women is increasing in different regions of the world [5, 11, 13, 14]. The most common antimicrobial resistance mechanisms are post-transcriptional methylation of adenine residues present in 23S rRNA, which is mediated by *erm* class gene-encoded methylases [15, 16], and efflux of the antibiotic mediated by a membrane-bound protein encoded by *mef* genes [17]. The expression of *erm* genes usually results in cross-resistance to macrolides, lincosamides and streptogramin B, the MLS_B_ phenotype [18]. On the other hand, resistance encoded by *mef* genes (phenotype M) confers resistance only to 14- and 15-membered ring macrolides (erythromycin and azithromycin) [19].

Standard culture-based methods for GBS detection involve the inoculation of a vaginal-rectal swab specimen into selective enrichment broth medium. Following enrichment, the specimen is subcultured on blood agar plates or alternatively on chromogenic Granada agar for visual detection of beta-hemolytic or orange carotenoid pigment-producing colonies, respectively. The identification of presumptive GBS colonies is performed by phenotypic methods. Moreover, it is also recommended that GBS isolated from penicillin-allergic pregnant women at risk of anaphylaxis should be screened for antimicrobial susceptibility pattern [8]. Corroborating this, a study of Desai and colleagues [20] reported that 8.8% of GBS-positive pregnant women also had a penicillin allergy at delivery.

In general, these procedures may require up to 72 h for results, which does not impact pregnant women undergoing routine prenatal care. However, many cases of GBS diseases have been reported in newborns from mothers with negative prenatal bacterial screen [21, 22]. These false-negative results may be due to limitations of the current culture methods that cannot promptly detect either non-hemolytic nor non-pigment producing isolates [23]. In addition, a small proportion of pregnant women may become colonized with GBS in the period following prenatal screening and the onset of labor [24]. Another concern associated with culture-based strategies is the unavailability of results for pregnant women in premature labor or who have not had prenatal care [25].

There is a need for a rapid and sensitive test for detecting GBS-colonized pregnant women at the time of delivery, and determining GBS antibacterial resistance to manage appropriate IAP. The aim of this study was to develop a melting curve-based multiplex real-time polymerase chain reaction (PCR) assay for simultaneous detection of GBS and macrolide and lincosamide resistance markers. The assay targets the *cfb* gene used for specific identification of GBS and *erm* and *mef* genes. The *cfb* gene encodes an extracellular pore-forming protein [26] known as CAMP (acronym for Christie, Atkins and Munch-Peterson) factor [27], which has been widely used for phenotypic identification of GBS isolates [28]. Furthermore, most nucleic acid amplification tests (including commercially available ones) target *cfb* gene for detection of GBS vaginal-rectal colonization [29]. The potential usefulness of the assay was evaluated for prenatal GBS screening in vaginal-rectal swab specimens. The results of the multiplex real-time PCR assay were compared with those obtained with culture-based analyses.

## Methods

### Microbial strains

A panel of 37 microbial species (27 bacteria and 10 fungi, Table 1) was used to develop the assays. These included various streptococcal and closed-related species and other microbial components of the intestinal and genital microbiota. Two species of *Cryptococcus* were also included. Reference strains were kindly donated by Instituto Oswaldo Cruz (FIOCRUZ, Rio de Janeiro, Brazil) and Laboratório Central do Paraná (LACEN, Paraná, Brazil). LMC and HU strains were obtained from the bacterial collection of the Laboratório de Microbiologia Clínica of the Universidade Estadual de Londrina (UEL); LBBA strains were obtained from the Laboratório de Bacteriologia Básica e Aplicada of UEL. Bacterial and fungal species were cultivated at 37 °C for 24 h in tryptic soy broth (TSB, Oxoid) and Sabouraud dextrose broth (SDB, Himedia), respectively. Bacteria and fungi were kept at − 20 °C in TSB containing 20% glycerol and 5% sheep blood and SDB containing 20% glycerol, respectively.

**Table 1:** Panel of microorganisms used to evaluate the multiplex real-time PCR specificity and sensitivity

Species	Source	Species	Source
*Streptococcus agalactiae*	ATCC 13813	*Escherichia coli*	ATCC 25922
*Streptococcus agalactiae*	LMC UEL 15	*Escherichia coli*	ATCC 35218
*Streptococcus agalactiae*	LMC UEL 65	*Klebsiella pneumoniae*	ATCC 700603
*Streptococcus agalactiae*	LMC UEL 66	*Proteus mirabilis*	HU-UEL
*Streptococcus agalactiae* serotype Ia	LMC UEL 43	*Providencia stuartii*	HU-UEL
*Streptococcus agalactiae* serotype II	LMC UEL 92	*Salmonella* sp.	HU-UEL
*Streptococcus agalactiae* serotype III	LMC UEL 59	*Shigella dysenteriae*	ATCC 13313
*Streptococcus agalactiae* serotype V	LMC UEL 73	*Enterococcus faecalis*	ATCC 29212
*Streptococcus agalactiae* serotype IX	LMC UEL 11	*Enterococcus faecium*	ATCC 6569
*Streptococcus dysgalactiae* subsp. *equisimilis* group G.	LACEN 6196	*Lactobacillus acidophilus*	ATCC 4356
*Streptococcus dysgalactiae* subsp. *equisimilis* group C	LACEN 53157	*Lactobacillus rhamnosus*	LBBA-UEL
*Streptococcus mitis*	ATCC 49456	*Lactococcus lactis* subsp. *lactis*	LBBA-UEL 22
*Streptococcus mutans*	ATCC 25175	*Lactococcus lactis* subsp*. cremoris*	LBBA-UEL 22–1
*Streptococcus pneumoniae*	ATCC 49619	*Leuconostoc mesenteroides*	LBBA-UEL 704
*Streptococcus pyogenes*	ATCC 19615	*Candida albicans*	ATCC 26790
*Streptococcus sanguis*	ATCC10557	*Candida bracarensis*	LMC UEL1217
*Staphylococcus aureus*	ATCC 25923	*Candida dubliniensis*	LMC UEL 947C
*Staphylococcus epidermidis*	ATCC 12228	*Candida glabrata*	LMC UEL 51B
*Staphylococcus haemolyticus*	ATCC 29668	*Candida metapsilosis*	LMC UEL 2263
*Staphylococcus saprophyticus*	HU-UEL	*Candida orthopsilosis*	LMC UEL 2259
*Bacillus subtilis*	ATCC 23857	*Candida parapsilosis*	ATCC 22019
*Aeromonas* sp.	HU-UEL	*Candida tropicalis*	ATCC 28707
*Pseudomonas aeruginosa*	ATCC 27853	*Cryptococcus gattii*	ATCC 56990
*Citrobacter freundii*	HU-UEL	*Cryptococcus neoformans*	ATCC 66031

*ATCC* American Type Culture Collection, *LMC* Laboratório de Microbiologia Clínica, *UEL* Universidade Estadual de Londrina, *LACEN* Laboratório Central do Estado do Paraná, *HU* Hospital Universitário de Londrina, *LBBA* Laboratório de Bacteriologia Básica e Aplicada

### DNA isolation from in vitro cultured microbial species

The Gentra Puregene Blood kit (Qiagen, Brazil) was used for DNA isolation, according to manufacturer’s recommendations. All clinical and reference strains were cultivated in specific broth medium at 37 °C for 24 h. Microbial cultures were centrifuged at 10,000 x *g* for 5 min, and the pellets were washed twice with sterile 0.15 M phosphate-buffered saline (PBS) pH 7.2 before DNA extraction.

### Oligonucleotide primers and PCR design

The nucleotide sequences of *cfb* encoding genes from *S. agalactiae* deposited in the GenBank/EMBL databases were analyzed using the *BioEdit v.7.2.0* software. Specific primers were designed using a consensus sequence and the OligoAnalyzer 3.1 (http://www.idtdna.com/calc/analyzer) tool. Primers for genes [*erm*(A) subclass of *erm*(TR)], *erm*(B) and *mef*(A/E) encoding erythromycin and clindamycin resistance were as described previously [5]. Primers targeting the human tRNA processing ribonuclease P (*RNAseP*) gene [30, 31] and intergenic spacer 1 (IGS1) of ribosomal RNA (rDNA) gene cluster of the *Cryptococcus gattii* [32], an encapsulated yeast found in the environment, were included in this study to evaluate the quality of the DNA and potential PCR interfering substances, respectively.

The primer sequences and expected size of amplicons are shown in Table 2. All primers were used in conventional PCR in a final volume of 25 μL containing 20 mM Tris-HCl, pH 8.4, 5 mM KCl, 1.5 mM MgCl_2_, 100 μM of each dNTP, 10 *p*mol of each forward and reverse primer, 2.5 U *Taq* DNA polymerase (Invitrogen, São Paulo, Brazil), and 2 *μ*L of genomic DNA. The amplification reactions were performed in a Veriti 96-well Thermal Cycler (Applied Biosystems) with an initial denaturation at 95 °C for 1 min, followed by 35 cycles of 95 °C for 30 s, annealing at 67 °C for 1 min and an extension step at 72 °C for 45 s. Negative template control (NTC) reactions without any template DNA were carried out simultaneously. Amplicons were analyzed by 3% agarose gel electrophoresis after DNA staining with 0.5 μg/mL ethidium bromide. The identity of the amplicons was confirmed after determination of the nucleotide sequences with a 3730 xl DNA Analyzer (Applied Biosystems) using the Big Dye Terminator v.3.1 Cycle Sequencing Kit. Search for homologies in the GenBank/EMBL databases was carried out with the Blast algorithm.

**Table 2:** Oligonucleotide primers of melting curve-based multiplex real-time PCR

Target^a^	Nucleotide sequence (5′ to 3′)	Amplicon size (bp)	Reference
*cfb* ^b^	F: CACACATGCTGTTGGAGTTCAGTTGA	138	This study
	R: ACGAAGTCGACAGCATCACACGAAA		
*erm*(A)/(TR)	F: CCGGCAAGGAGAAGGTTATAATGA	190	Otaguiri et al. [5]
	R: GCATTCACCCGTTGACTCATTTCC		
*erm*(B)	F: GCTCTTGCACACTCAAGTCTCGAT	117	Otaguiri et al. [5]
	R: ACATCTGTGGTATGGCGGGTAAGT		
*mef*(A/E)	F: GCGATGGTCTTGTCTATGGCTTCA	225	Otaguiri et al. [5]
	R: AGCTGTTCCAATGCTACGGAT		
*RNaseP*	F: AGATTTGGACCTGCGAGCG	64	WHO [27]
	R: GAGCGGCTGTCTCCACAAGT		
IGS1	F: GTCATTTCAGCTGGCGCCATCGATAC	260	Tavares et al. [32]
	R: TTGCCGCATAACGCATCTTAGCCA		

^a^*cfb* gene encodes the CAMP factor; *erm* genes encode 23S rRNA methylases; *mef* gene encodes efflux pumps; *RNaseP* gene encodes human ribonuclease P; IGS1, intergenic spacer 1 of ribosomal RNA gene cluster of *Cryptococcus gattii*. ^b^The nucleotide sequences of *Streptococcus agalactiae* genes deposited in the GenBank/EMBL databases were used for specific primer design

### Multiplex real-time PCR assay

All PCRs were performed on a Rotor-Gene Q 5-Plex (Qiagen, Germany), and the assay conditions were optimized for various parameters, including concentration of each primer set, annealing temperature and number of PCR cycles (data not shown). The optimized assay was performed in two separate tubes each containing a final volume of 25 μL: a) 2× High-Resolution Melt (HRM) PCR Master Mix (Qiagen, Brazil), 10 *ρ*mol of forward and reverse *erm*(B), *cfb* and IGS1 primer sets, 20 *ρ*mol of forward and reverse *mef*(A/E) primers, and 10 *n*g of recombinant plasmid pCR2.1/IGS1 [32]; b) 2× HRM PCR Master Mix and 10 *ρ*mol of forward and reverse *erm*(A) and human *RNAseP* primers. For both reaction mixtures, 6 *μ*L of template DNA were added and the final volume was adjusted with deionized water. The cycling conditions included an initial denaturation step at 95 °C for 5 min, followed by 35 cycles of 95 °C for 10 s, annealing at 67 °C for 30 s and an extension step at 72 °C for 20 s. Melting curves were acquired using 0.05 °C steps with a hold of 60 s at each step from 75 to 85 °C. NTC reactions were carried out simultaneously. Data were analyzed using Rotor Gene software version.

### Analytical specificity and sensitivity

Multiplex real-time PCR specificity was analyzed using 100 *η*g genomic DNA obtained from cultures of a panel of bacteria and fungi (Table 1). All amplification reactions were performed in duplicate in three independent experiments*.* In silico analysis was also carried out to determine the specificity of the *cfb* amplification reactions. Primer sequences targeting the *cfb* gene were compared with nucleotide sequences available in GenBank databases of the National Center for Biotechnology Information (NCBI, http://www.ncbi.nlm.nih.gov) using the Blast algorithm (*blastn*).

Multiplex real-time PCR sensitivity was determined empirically using macrolide and lincosamide resistant GBS strains (LMC UEL 15 *cfb*^*+*^, *mef*(A/E)^*+*^; LMC UEL 65 *cfb*^*+*^, *erm*(A)^*+*^; LMC UEL 66 *cfb*^*+*^, *erm*(B)^*+*^), according to published recommendations [33]. Three colonies forming unit (CFU) of each GBS strains were cultivated in TSB at 37 °C for 24 h. The bacterial cells were harvested by centrifugation (10,000 x *g* for 5 min), washed twice with sterile PBS and the cell density was adjusted to 9.0 × 10^8^ (3.0 McFarland standard) using the DensiCHECK™ PLUS colorimeter (bioMérieux, Brazil) in 1.0 mL of the same buffer. DNA extraction was performed as above. Each strain was processed in duplicate on five consecutive days. Tenfold serial dilutions were prepared and 6 μL of DNA template of each dilution were included in amplification reactions. For each primer pair, a standard curve was generated from the Ct values as a function of log CFU and R^2^ was calculated to evaluate the efficiency of the reaction. The slope of this line was used to determine the efficiency (E) according to the equation: *E* = 10^–1/slope^ – 1.

### Performance of the multiplex real-time PCR assay in clinical samples in comparison to culture based analysis

The performance of real-time PCR in GBS isolates and clinical samples was compared with the results obtained from microbiological analyses. Thirty one GBS isolates recovered from the vaginal-rectal swab screening cultures of women seen at University Hospital of Londrina, Paraná, Brazil from March to September of 2012 [5] were analyzed by the multiplex real-time PCR assay. These isolates were taken from the bacterial collection of the Laboratory of Clinical Microbiology of Universidade Estadual de Londrina and were processed as described below. For direct analysis, a total of 102 pregnant women seen at the UniversityHospital of Londrina, Paraná, Brazil from June to December 2015, and October 2017 were enrolled in this study. The study protocol was approved by the Ethics Committee of the Universidade Estadual de Londrina (Document 193/12-CEP/UEL). Written informed consent was obtained from the women to participate in this study, agreeing with the publication of this report and any accompanying images. Two vaginal-rectal swabs of each woman were collected. Sampling was performed on the lower third of the vagina followed by the rectum using the COPAN Transystem Stuart collection device (COPAN Diagnostic, Italy).

One swab specimen was inoculated into a Granada Biphasic broth (BioMérieux, Brazil) and incubated at 37 °C for 24 h. After incubation, the sample was subcultured on Columbia blood agar base (Oxoid, Brazil) containing 5% sheep blood (Newprov, Brazil) at 37 °C for 24 h. Suggestive colonies of GBS were subjected to standard phenotypic identification based on colony morphology, Gram staining, catalase and CAMP tests. Concomitantly, tests for growth in 6.5% NaCl, bile-esculin reaction, sodium hippurate hydrolysis, and susceptibility to bacitracin and sulfamethoxazole *plus* trimethoprim were also performed. Bacteria were kept at − 20 °C in TSB containing 20% glycerol and 5% sheep blood. The second swab was vortexed for 2 min in 1 mL of deionized sterile water, the suspension was centrifuged and the pellet was used for DNA extraction as described above. DNA was stored at − 20 °C until use.

GBS isolates were tested for penicillin, clindamycin and erythromycin susceptibility using the disk-diffusion method according to the recommendations of the Clinical Laboratory Standards Institute [34]. The phenotypes of erythromycin- and clindamycin-resistant GBSs were determined by the double-disk diffusion method as described by Seppala et al. [35].

## Results

### Assay design

**Fig. 1:**
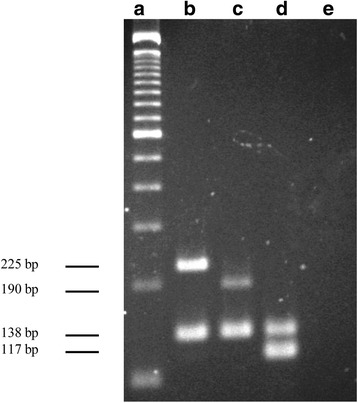
Multiplex PCR assay for simultaneous detection of cfb and erythromycin and clindamycin resistance-encoding genes in conventional PCR. **a** 100-bp molecular size ladder; (**b**) LMC UEL 15 (*mef*(A/E) and *cfb*); (**c**) LMC UEL 65 (*erm*(A) and *cfb*); (**d**) LMC UEL 66 (*erm*(B) and *cfb*); (**e**) negative template control

**Fig. 2:**
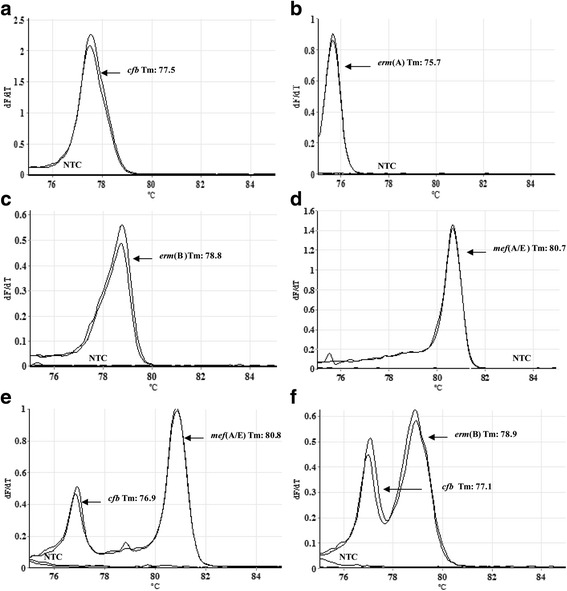
Melting curve analysis showing the melting temperature peaks (Tm) of *Streptococcus agalactiae* with macrolide and lincosamide resistance genes and negative template controls (NTC). **a** LMC UEL 65 (*cfb*); (**b**) LMC UEL 65 [*erm*(A)]; (**c**) LMC UEL 66 [*erm*(B)]; (**d**) LMC UEL 15 [*mef*(A/E)]; (**e**) LMC UEL 15 [*cfb* and *mef*(A/E)]; (**f**) LMC UEL 66 [*cfb* and *erm*(B)]

In this study, a multiplex-PCR assay using real-time and melting curves was standardized for simultaneous detection of the genes *cfb*, *erm*(A), *erm*(B), and *mef*(A/B). The conditions of amplification for simultaneous detection of these genes were first standardized in conventional monoplex PCR using genomic DNA of GBS strains. All specific primer pairs generated amplicons with the expected size shown in Table 2 using an annealing temperature of 67 °C. The identity of each amplicon was further confirmed by sequencing and searching for nucleotide sequence homology in the GenBank/EMBL databases. After determining the optimal conditions for amplification, all primer pairs were combined in a conventional multiplex-PCR format and the results are shown in Fig. 1. For establishment of melting-curve based multiplex real-time PCR, equivalent melting temperatures (T_m_) of each primer pair were initially detected in a monoplex real-time PCR assay. All primer pairs successfully amplified the corresponding genes generating a dissociation curve with a single peak, and the T_m_ values of all amplicons were as follows: 76.7 ± 0.4 °C for *cfb*, 75.5 ± 0.5 °C for *erm*(A), 78.8 ± 0.7 °C for *erm*(B), 80.65 ± 0.55 °C for *mef*(A/E) (Fig. 2). In addition, T_m_ values of 82.8 ± 0.55 °C and 81.8 ± 0.21 °C were detected for *RNaseP* gene and IGS1 region, respectively (Additional file 1: Figure S1A-B). According to these data, the multiplex real-time PCR assay was performed with two tubes in one reaction. One tube corresponded to the targets *cfb*, *erm*(B), *mef*(A/E) genes, IGS1 region and recombinant plasmid pCR2.1/IGS1, and the other to the targets *erm*(A) and *RNaseP* genes.

### Analytical performance

The specificity of multiplex real-time PCR was determined using genomic DNA from a panel of bacteria and fungi (Table 1), and amplification signals were detected for all GBS strains, including five different capsular serotypes. No cross-reactivity was observed between non-GBS strains. Primer specificity for the *cfb* gene was also evaluated in silico using the GenBank/EMBL database of the NCBI homepage, and no matches were found other than those with the corresponding gene of GBS.

**Fig. 3:**
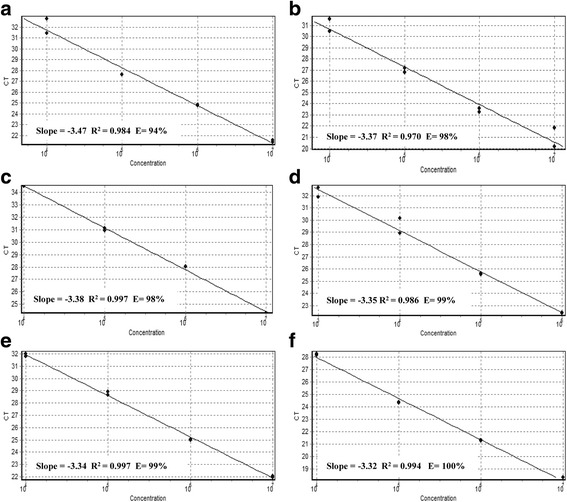
Sensitivity of multiplex real-time PCR assays. **a** LMC UEL 65 (*cfb*); (**b**) LMC UEL 65 [*erm*(A)]; (C) LMC UEL 66 [*erm*(B)]; (**d**) LMC UEL 15 [*mef*(A/E)]; (**e**) LMC UEL 15 [*cfb* and *mef*(A/E)]; (**f**) LMC UEL 66 [*cfb* and *erm*(B)]. Amplification plot of 10-fold serial dilution corresponding to 10^4^–10^7^ CFU; standard curve represented by linear regression line for threshold cycle (Ct) versus sample log concentration. Slope, regression coefficient and efficiency of the real-time PCR method are noted (**a**-**f**)

The linearity and limits of detection (LOD) of the multiplex real-time PCR for the target DNAs were determined with tenfold serial dilutions (at a cell density of 10^7^ to 10 CFU equivalents per reaction) of each genomic DNA from macrolide- and lincosamide-resistant GBS strains. Each concentration was analyzed in 6 replicates on five different days (*n* = 30). The LOD of the multiplex real-time PCR for the target DNAs was 10^4^ CFU equivalents per reaction, and the reaction efficiencies calculated from the slope of the standard curve were within the range of 94 to 100% (Fig. 3).

To further verify the specific performance of the assay, genomic DNA extracted from 31 GBS isolates from the bacterial collection were analyzed by the multiplex real-time PCR. The results showed 100% concordance with those obtained previously by Otaguiri et al. [5]. All isolates were positive for *cfb* gene and the erythromycin and lincosamide resistance markers were detected in three isolates: one isolate each carried the *mef*(A/E); *erm*(B); and *erm*(A) and *erm*(B) genes.

### Evaluation of real-time multiplex PCR in clinical samples

Performance of the multiplex real-time PCR assay was analyzed in vaginal-rectal swabs obtained from 102 pregnant women, and the results were compared to the standard culture-based method for GBS detection. *RNaseP* and IGS1 amplification signals were detected in all reactions, indicating no PCR inhibitors (Additional file 1: Figure S1A-B). NTC amplification signals were not detected in any specific PCR. The prevalence of GBS colonization among the population studied was 15.7% (16/102) based on a positive culture and the multiplex real-time PCR results. Agreement between the two assays was found for 11 (68.75%) GBS-colonized women. Four samples (25%) were positive by multiplex real-time PCR and negative by the culture method, and one (6.25%) was negative by multiplex real-time PCR and positive by the culture method.

Using the culture-based results as a reference, the multiplex real-time PCR had a sensitivity of 91.7% (11/12, CI 59.7–99.5%), a specificity of 95.5% (86/90, CI 88.4–98.6%), a positive predictive value of 73.3% (11/15, CI 44.8–91.1%) and a negative predictive value of 98.9% (86/87, CI 92.9–99.9%) (Table 3).

**Table 3:** Sensitivity, specificity, positive predictive value (PPV) and negative predictive value (NPV)

Multiplex real-time PCR	Culture^a^		Total
	Positive	Negative	
Positive	11	4	15
Negative	1	86	87
Total	12	90	102
Sensitivity (95% CI)^b^	91.7% (59.7–99.5%)		
Specificity (95% CI)^b^	95.5% (88.4–98.6%)		
PPV (95% CI)^b^	73.3% (44.8–91.1%)		
NPV (95% CI)^b^	98.9% (92.9–99.9%)		

^a^Standard routine culture of vaginal-rectal swab specimen collected from pregnant women at 35–37 weeks of gestation; ^b^Values calculated with 95% confidence interval (CI) using the program available at http://faculty.vassar.edu/lowry/clin1.html

All GBS isolates were susceptible to penicillin according to the disk-diffusion method. Regarding the erythromycin and clindamycin susceptibility profile, of the 11 GBS-positive isolates by the culture method, 10 were susceptible and one was resistant to both antibacterials according to the phenotypic methods. Of the four vaginal-rectal swabs testing positive for GBS by multiplex real-time PCR, one was negative for antimicrobial resistance markers, two tested positive for *erm*(B) and *mef*(A/E) genes, and the other tested positive for *mef*(A/E) genes.

For the comparison analysis, the GBS-colonized pregnant women whose *cfb* gene was not detected by multiplex real-time PCR or tested negative in culture approaches were excluded from the comparative analysis. The phenotypic results were in accordance with those obtained in real-time multiplex PCR for nine GBS-colonized pregnant women. No antimicrobial resistance marker was detected in seven susceptible isolates. One erythromycin/clindamycin-resistant isolate displayed the constitutive macrolide-lincosamide-streptogramin B (cMLS_B_) phenotype, and carried the *erm*(A) and *erm*(B) genes. Whereas, one erythromycin-resistant isolate carried the *mef*(A/E) gene. In the discordant results, two pregnant women colonized with erythromycin/clindamycin susceptible isolates, one tested positive for *erm*(A) and *mef*(A/E) and the other for *erm*(B) genes by the multiplex real-time PCR assay (Table 4).

**Table 4:** Data from phenotypic characterization and multiplex real-time PCR of *Streptococcus agalactiae*

Isolates	Susceptibility phenotype		Real-time multiplex PCR			
	E^a^	DA^b^	*cfb* ^c^	*erm*(A)^c^	*erm*(B)^c^	*mef*(A/E)^c^
LMC UEL 5	S	S	+	–	–	–
LMC UEL 21	S	S	+	–	–	–
LMC UEL 23	S	S	+	–	+	–
LMC UEL 27	S	S	+	–	–	–
LMC UEL 30	S	S	+	–	–	–
LMC UEL 34	S	S	+	–	–	–
LMC UEL 43	S	S	+	–	–	–
LMC UEL 43A	R	R	+	+	+	–
LMC UEL 57	S	S	+	+	–	+
LMC UEL 60	S	S	+	–	–	–
LMC UEL 68	S	S	–	–	–	–
LMC UEL 103	R	S	+	–	–	+
LMC UEL 28	CN	CN	+	–	–	–
LMC UEL 63	CN	CN	+	–	+	+
LMC UEL 95	CN	CN	+	–	–	+
LMC UEL 99	CN	CN	+	–	+	+

^a^E (erythromycin) and ^b^DA (clindamycin) resistance phenotypes were determined by the double-disk diffusion method [34]. (S) Susceptible; (R) Resistant; (CN) Culture-Negative. ^c^Target genes detected in vaginal-rectal swab specimens by multiplex real-time PCR. (+) Presence; (−) Absence

All GBS isolates were subjected to re-examination using genomic DNA extracted from axenic cultures and there was no difference between the concordant results. For the above mentioned two discrepant results, the *erm(*A), *erm*(B) and *mef*(A/E) genes were not detected by multiplex real-time PCR, confirming the phenotypic results, and indicating the presence of other bacteria carrying the detected genes in the vaginal-rectal swab sample.

## Discussion

Real-time PCR is one of the rapid and feasible methods for maternal intrapartum GBS screening, and most of the in house and commercial tests are based on the utilization of probes [29, 36–41]. In this study, a sensitive melting curve-based multiplex real-time PCR was designed and evaluated for simultaneous detection of GBS and the most prevalent macrolide and lincosamide resistance markers. According to the literature, only the study of Dela Cruz et al. [42] reported an assay for simultaneous detection of GBS and antimicrobial resistance markers. These authors developed a probe-based real-time multiplex PCR for detection of *cfb*, *erm*(TR), *erm*(B) and *mef*(A/E) genes in genomic DNA extracted from GBS cultures isolated from vaginal-rectal swabs, with a sensitivity of 93% and specificity of 90%.

The analytical and experimental data showed that the primers designed, in this study, to target the GBS *cfb* gene did not cross-react with another nucleotide sequence of different microbial species. One GBS-colonized pregnant woman was falsely identified as a non-GBS carrier by multiplex real-time PCR. In this case, the presence of PCR inhibitors was discarded since the amplification signals of the *RNaseP* and IGS1 controls were detected in the reaction. Thus, this result could be explained by the low bacterial load on the swab, which was below the LOD of the assay. Similarly, other real-time PCR-based assays for GBS detection have shown discrepant results when compared to culture-based approach [43], including those marketed tests [36].

Several real-time PCR-based assays have been developed in the last decades for GBS detection in vaginal-rectal swab from pregnant women [29, 36–41]. Most of these studies target the *cfb* gene for specific detection of GBS [8, 29, 37]. However, the following genes were also used for GBS detection by real-time PCR assays: those of the operon *dlt* [37, 38], which catalyze the incorporation of D-alanine residues into GBS cell wall lipoteichoic acids [44]; *cyl*B [39], which encodes a transmembrane protein of ABC transporter required for the production of GBS hemolysin [45]; *ssr*A [40], encoding tmRNA involved in the degradation of truncated proteins [46]; and *sip* [41], encoding a surface immunogenic protein [47].

The timely direct detection of resistance genes in GBS from pregnant women will contribute to prompt and appropriate administration of antimicrobial during the intrapartum period. In addition, the IAP for prevention of GBS neonatal infections has raised worries about the selection of antimicrobial resistant and/or potentially more virulent microorganisms for newborns [10]. Since the *erm* genes are located mainly on mobile genetic elements such as plasmids and conjugative transposons [16, 48], selective pressure imposed by the antimicrobials may trigger horizontal DNA transfer between microbiota members, contributing to the spread of resistance. Thus, besides reliable GBS detection, the determination of its antimicrobial susceptibilities is important to implement effective IAP for all GBS-colonized pregnant women, thereby preventing inappropriate use of antimicrobials.

In this study, a good agreement was observed between culture- and PCR-based results for GBS positive result and the presence of erythromycin and clindamycin resistance encoded genes. However, two false-positive were detected by multiplex real-time PCR regarding the resistance markers. In fact, other bacterial species colonizing the urogenital and intestinal tracts that are known to harbor *erm* and *mef* genes [49] can be detected in a molecular assay. Taken together, the data indicate that these genes are not suitable for specific detection of GBS resistance markers in direct analysis of vaginal-rectal swabs. Despite this limitation, the assay for simultaneous detection of GBS and resistance markers could reduce the turnaround time (about 24–48 h) for both GBS identification and detection of its antimicrobial resistance after axenic cultivation compared to phenotypic methods. Moreover, in smaller laboratories with limited resources due to the equipment cost and the price of a single real-time PCR test, this assay could be used in a conventional multiplex-PCR format, which can be performed in one tube.

In general, nucleic acid amplification tests (NAAT) have been proven highly specific and with higher sensitive (reported sensitivity range of 86 to 100%) for detection of GBS vaginal-rectal colonization compared to the conventional culture-based test [8, 29, 36, 50]. This difference can be due to the presence of both non-viable cells or GBS antagonistic microorganisms [51]. In this study, the number of vaginal-rectal swabs from pregnant women analyzed can limit generalization. More samples may provide better estimates with less uncertainty [33]. Despite this limitation, the clinical sensitivity and specificity of the multiplex real-time PCR assay were determined to be 91.7 and 95.1%, respectively, which was comparable to the results of previously reported studies based on probe approaches [29, 36–41].

Although a number of commercial kits are available, their utilization has not yet been universally implemented in hospitals, primarily due to costs and inability to determine the antimicrobial susceptibility profile if a NAAT shows positive. As stated before, most of the real-time PCR assays previously described (including those commercially available ones) use a specific probe for the target gene, besides oligonucleotide primers, which increases the costs [29, 36–41].

In this study, labor costs (the primers for resistance markers, equipment and personal were not included) for sample collection and processing of multiplex real-time PCR were estimated at US$3.47, compared with culture screening estimated cost of US$4.95 *per* swab. Furthermore, the assay provided a short turnaround time as full test, including DNA extraction, sample preparation and multiplex real-time PCR analysis, which can be performed in about 4 h. Another limitation of this study is that the time between vaginal-rectal swab collection and delivery was not analyzed and it was not possible to evaluate whether the result could be available in time for IAP. However, this assay provides reliable and faster results than culture that will help make appropriate decisions about the administration of antibiotics for neonates of women with unknown GBS colonization *status*.

## Conclusion

The results presented here showed that the multiplex real-time PCR is a rapid, affordable and sensitive assay suitable for direct detection of GBS in vaginal-rectal swab. Accordingly, the present molecular assay has potential usefulness during the intrapartum period, mainly for women who did not have a prenatal screening result. In the present format, simultaneous detection of GBS and its erythromycin and lincosamide resistance markers should be applied after bacterium recover by cultivation.

## Additional file

Additional file 1: **Figure S1.** Melting curve analysis showing the melting temperature peaks (Tm) of *RNaseP* (A) and IGS1 (B) controls. Primers targeting the human tRNA processing ribonuclease P (*RNAseP*) gene and intergenic spacer 1 (IGS1) of ribosomal RNA (rDNA) gene cluster of the *Cryptococcus gattii*, an encapsulated yeast found in the environment, were included in this study to evaluate the quality of the DNA and potential PCR interfering substances, respectively. The multiplex real-time PCR assay was performed with two tubes in one reaction using a Rotor-Gene Q 5-Plex equipment (Qiagen, Germany): a) 2× High-Resolution Melt (HRM) PCR Master Mix (Qiagen, Brazil), 10 *ρ*mol of forward and reverse *erm*(B), *cfb* and IGS1 primer sets, 20 *ρ*mol of forward and reverse *mef*(A/E) primers, and 10 *n*g of recombinant plasmid pCR2.1/IGS1 [32]; b) 2× HRM PCR Master Mix and 10 *ρ*mol of forward and reverse *erm*(A) and human *RNAseP* primers. The cycling conditions included an initial denaturation step at 95 °C for 5 min, followed by 35 cycles of 95 °C for 10 s, annealing at 67 °C for 30 s and an extension step at 72 °C for 20 s. Melting curves were acquired using 0.05 °C steps with a hold of 60 s at each step from 75 to 85 °C. NTC reactions were carried out simultaneously. Data were analyzed using Rotor Gene software version. (TIF 330 kb)

## Abbreviations

ATCC: American Type Culture Collection
CAMP: acronym for Christie, Atkins and Munch-Peterson
CFU: Colonies Forming Unit
CI: Confidence Interval
GBS: Group B *Streptococcus*
HRM: High-Resolution Melt
IAPS: Intrapartum Antimicrobial Prophylaxis
LBBA: Laboratório de Bacteriologia Básica e Aplicada
LMC: Laboratório de Microbiologia Clínica
LOD: Limits of Detection
NAAT: Nucleic Acid Amplification Test
NTC: Negative Template Control
PCR: Polimerase Chain Reaction
UEL: Universidade Estadual de Londrina
