# Supplementary figures and images for: Development of a melting-curve based multiplex real-time PCR assay for simultaneous detection of Streptococcus agalactiae and genes encoding resistance to macrolides and lincosamides
Source: BMC Pregnancy Childbirth. 2018 May 3;18:126. doi: 10.1186/s12884-018-1774-5 (PMC5934892; doi:10.1186/s12884-018-1774-5)

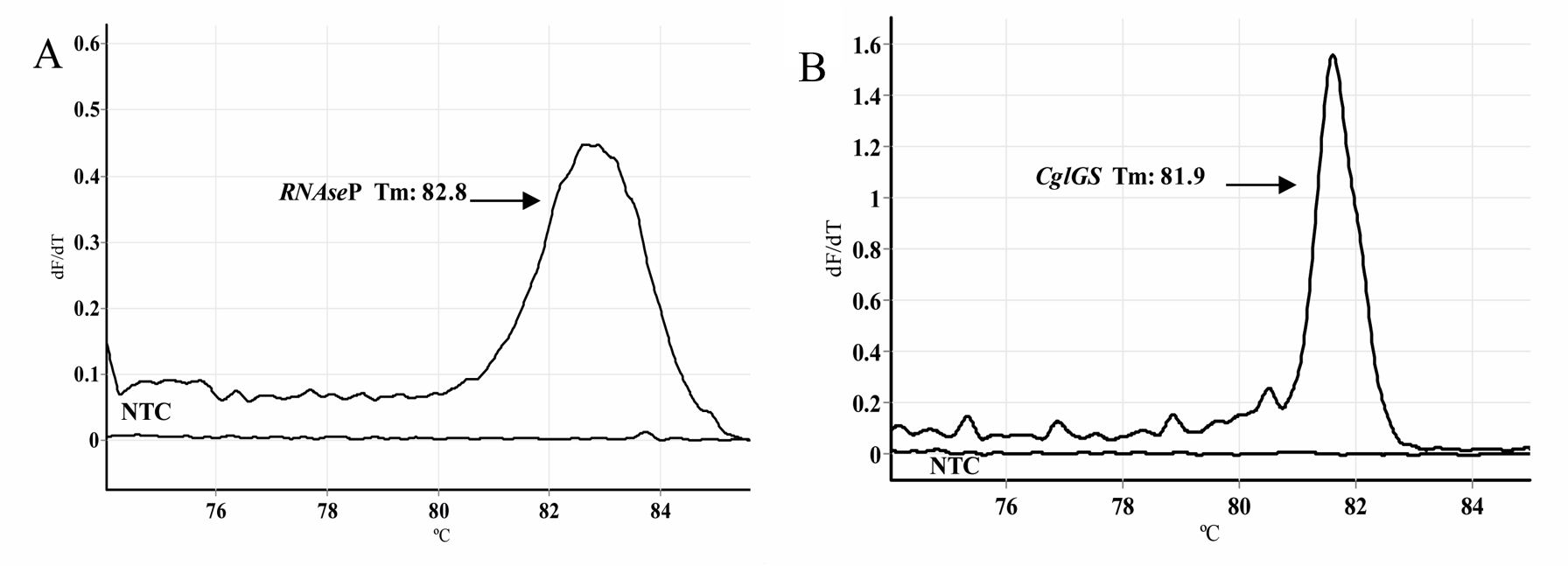

Supplement: Supplementary file 1 — Figure S1. Melting curve analysis showing the melting temperature peaks (Tm) of RNaseP (A) and IGS1 (B) controls. Primers targeting the human tRNA processing ribonuclease P (RNAseP) gene and intergenic spacer 1 (IGS1) of ribosomal RNA (rDNA) gene cluster of the Cryptococcus gattii, an encapsulated yeast found in the environment, were included in this study to evaluate the quality of the DNA and potential PCR interfering substances, respectively. The multiplex real-time PCR assay was performed with two tubes in one reaction using a Rotor-Gene Q 5-Plex equipment (Qiagen, Germany): a) 2× High-Resolution Melt (HRM) PCR Master Mix (Qiagen, Brazil), 10 ρmol of forward and reverse erm(B), cfb and IGS1 primer sets, 20 ρmol of forward and reverse mef(A/E) primers, and 10 ng of recombinant plasmid pCR2.1/IGS1 [32]; b) 2× HRM PCR Master Mix and 10 ρmol of forward and reverse erm(A) and human RNAseP primers. The cycling conditions included an initial denaturation step at 95 °C for 5 min, followed by 35 cycles of 95 °C for 10 s, annealing at 67 °C for 30 s and an extension step at 72 °C for 20 s. Melting curves were acquired using 0.05 °C steps with a hold of 60 s at each step from 75 to 85 °C. NTC reactions were carried out simultaneously. Data were analyzed using Rotor Gene software version. (TIF 330 kb) [file 12884_2018_1774_MOESM1_ESM.tif]
